# The VEGF inhibitor vatalanib regulates AD pathology in 5xFAD mice

**DOI:** 10.1186/s13041-020-00673-7

**Published:** 2020-09-25

**Authors:** Seong Gak Jeon, Hyun-ju Lee, HyunHee Park, Kyung-Min Han, Hyang-Sook Hoe

**Affiliations:** Department of Neural Development and Disease, Korea Brain Research Institute (KBRI), 61, Cheomdan-ro, Dong-gu, Daegu, 41062 Republic of Korea

**Keywords:** Alzheimer’s disease, Tau, Amyloid beta, 5xFAD mice, Vatalanib, Vascular endothelial growth factor, Tyrosine kinase inhibitor

## Abstract

Alzheimer’s disease (AD) is a highly prevalent neurodegenerative disease characterized by Aβ accumulation and tau hyperphosphorylation. Epidemiological evidence for a negative correlation between cancer and AD has led to the proposed use of tyrosine kinase inhibitors (TKIs) such as dasatinib and masitinib for AD, with reported beneficial effects in the AD brain. The TKI vatalanib inhibits angiogenesis by inhibiting vascular endothelial growth factor receptor (VEGFR). Although changes in VEGF and VEGFR have been documented in AD, the effect of vatalanib on AD pathology has not been investigated. In this study, the effects of vatalanib on tau phosphorylation and Aβ accumulation in 5xFAD mice, a model of AD, were evaluated by immunohistochemistry. Vatalanib administration significantly reduced tau phosphorylation at AT8 and AT100 by increasing p-GSK-3β (Ser9) in 5xFAD mice. In addition, vatalanib reduced the number and area of Aβ plaques in the cortex in 5xFAD mice. Our results suggest that vatalanib has potential as a regulator of AD pathology.

Alzheimer’s disease (AD) is the most common progressive neurodegenerative disease. The accumulation of amyloid β (Aβ) plaques and neurofibrillary tangles (NFTs) in the brain is the neuropathological hallmark of AD. Aβ plaques are formed from the accumulation of Aβ produced by sequential cleavage of amyloid precursor protein (APP) via β- and γ-secretase, and NFTs are aggregates of hyperphosphorylated tau, which normally functions in microtubule stabilization. Aβ plaque and NFT levels are both discriminators of and contributors to AD progression and are closely correlated with cognitive impairment in AD subjects [1]. Although AD and cancer share aging as a common factor, accumulating epidemiological evidence indicates a negative correlation between cancer and AD. This evidence has led to the proposed repurposing of cancer drugs of various mechanisms of action for the treatment of AD [2]. These cancer drugs include tyrosine kinase inhibitors (TKIs) such as dasatinib, masitinib, and imatinib, which have been shown to have therapeutic effects on the pathogenesis of AD [3–5].

Vatalanib, a small-molecule anticancer drug that inhibits angiogenesis (Fig. 1a), is a broad-spectrum TKI that exerts its effects by occupying the ATP-binding sites of vascular endothelial growth factor receptor 1–3 (VEGFR1–3), platelet-derived growth factor receptor α (PDGFRα), and c-KIT [6]. VEGF and VEGFR have been implicated in angiogenesis as well as blood–brain-barrier (BBB) permeability and microglial chemotaxis in AD pathology [7–9]. VEGF levels are increased in the peripheral blood, cerebrospinal fluid, and microglia of patients with AD and are correlated with the clinical severity of AD [8, 10, 11], and VEGF and VEGFR expression are also increased in AD animal models [12]. However, the effect of vatalanib on tau phosphorylation and Aβ accumulation in the AD brain has not been studied.

**Fig. 1:**
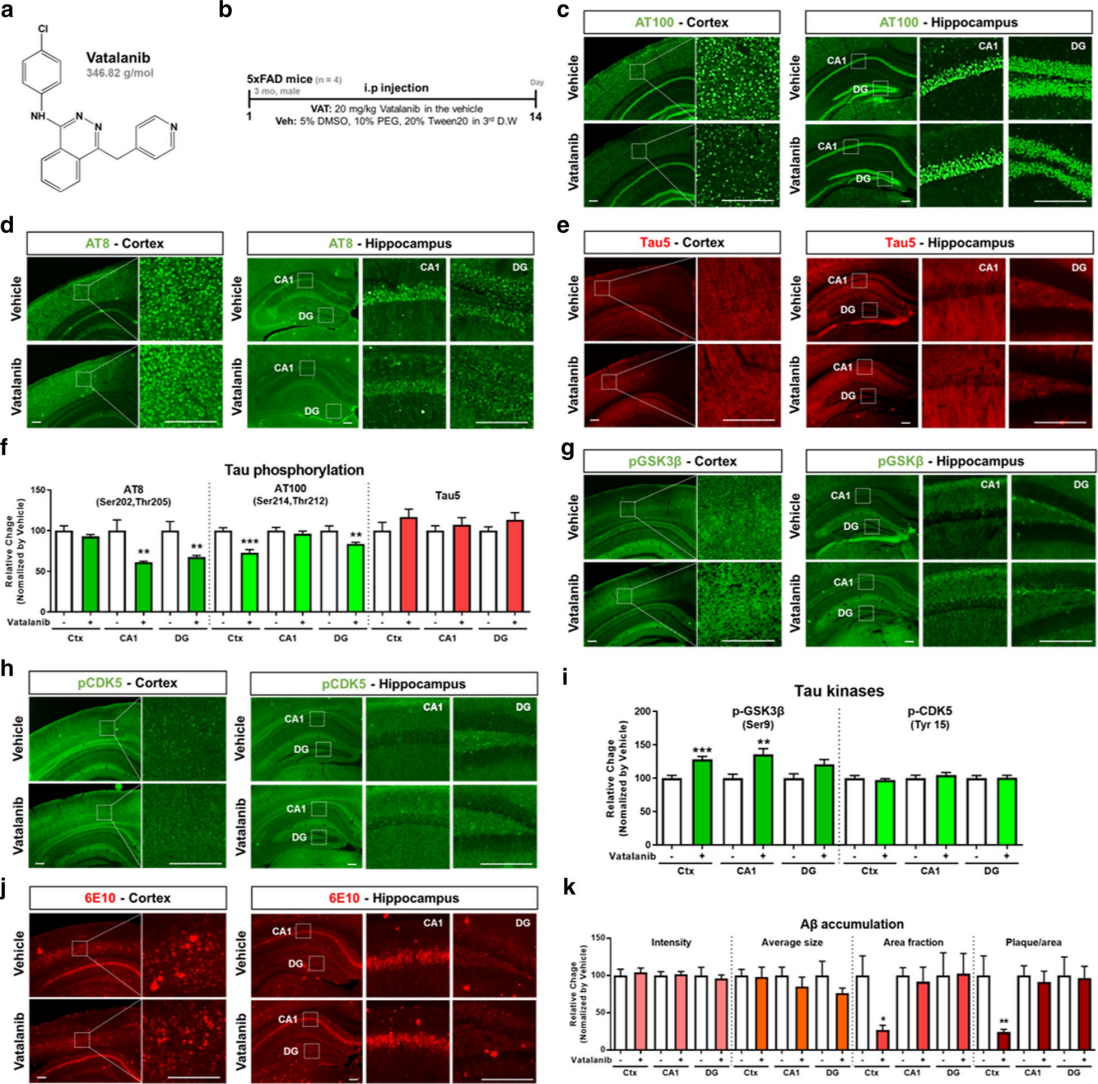
Changes in tau phosphorylation and Aβ plaque levels in vatalanib-injected 5xFAD mice. **a** Molecular structure and weight of vatalanib. **b** 3-month-old male 5xFAD mice were intraperitoneally injected with vehicle (Veh) or vatalanib (VAT; 20 mg/kg) daily for 14 days and subsequently sacrificed for histological analysis (see Materials and Methods in Additional file 1). **c**–**f** Representative images of immunohistochemical staining with tau phosphorylation-related antibodies in vatalanib-injected 5xFAD mice: anti-AT8 (**c**), anti-AT100 (**d**),and anti-Tau-5 (**e**) (n = 4 mice/group). **g**–**i** Representative images of immunohistochemical staining with tau kinase-related antibodies in vatalanib-injected 5xFAD mice: anti-p-GSK-3β (**g**) and anti-p-CDK5 (**h**) (n = 4 mice/group). **j** Representative images of immunohistochemical staining with an anti-6E10 antibody in vatalanib-injected 5xFAD mice. **k** Quantification of Ab intensity, average size, area fraction, and number of Ab plaques per area from (**j**) (n = 4 mice/group). All histological quantification results for the vatalanib-treated group (+) were normalized by the vehicle-treated group (−). Scale bar = 200 μm. *p < 0.05; *p < 0.01; ***p < 0.001 vs vehicle-treated group. N = number of groups

To investigate the effect of vatalanib on the AD brain, we administered 20 mg/kg of vatalanib daily for 14 days to 3-month-old 5xFAD mice, a model of AD (Fig. 1b). Eight hours after the last injection, the mice were fixed via sequential cardiac perfusion with PBS and 4% paraformaldehyde, and immunofluorescence staining was performed using AT100 (Ser^202^ and Thr^205^), AT8 (Ser^214^ and Thr^212^), and Tau5 (total tau) antibodies to visualize tau phosphorylation (Fig. 1c–e). Histological quantification of the fluorescence intensity of tau phosphorylation (Fig. 1f) revealed a significant decrease in AT8 immunoreactivity in the hippocampus (CA1 and DG) of vatalanib-treated 5xFAD mice compared with vehicle-treated 5xFAD mice (Fig. 1c, f). The fluorescence intensity of AT100 was also significantly decreased in the cortex and hippocampus DG of vatalanib-treated 5xFAD mice (Fig. 1d, f). However, the fluorescence intensity of Tau5 was not altered by the administration of vatalanib (Fig. 1e, f). Thus, vatalanib administration in 5xFAD mice significantly reduced phospho-tau^Ser202, Thr205^ and phospho-tau^Thr212, Ser214^ without altering total tau levels.

To investigate the molecular mechanism by which vatalanib modulates tau phosphorylation in 5xFAD mice, immunofluorescence staining was performed with antibodies against p-GSK-3β and p-CDK5, the major molecules involved in tau phosphorylation (Fig. 1g, h). Histological quantification of the fluorescence intensity of tau kinases (Fig. 1i) revealed a significant increase in p-GSK-3β^Ser9^ in the cortex and hippocampus CA1 of vatalanib-administered 5xFAD mice (Fig. 1g, i). However, another major tau kinase, p-CDK5, was not altered by the administration of vatalanib in 5xFAD mice (Fig. 1h, i). It is well known that the constitutive activity of GSK-3β is inhibited by GSK-3β (Ser9) phosphorylation. Therefore, our results suggest that increased phosphorylation of GSK-3β (Ser9) may have contributed to the reduction of tau phosphorylation by vatalanib administration.

To investigate the accumulation of Aβ, another histopathological feature of AD, immunofluorescence staining was performed in vatalanib-treated mice using a 6E10 antibody that labels Aβ_1–16_ as an epitope (Fig. 1j). To evaluate the effect of vatalanib on Aβ accumulation in 5xFAD mice, the fluorescence intensity, area fraction and average size of Aβ plaques and the number of Aβ plaques per area were quantified based on 6E10 immunoreactivity (Fig. 1k). Although vatalanib administration did not alter the fluorescence intensity of Aβ plaques in 5xFAD mice, the area fraction of Aβ plaques in the cortex was significantly reduced. The number of Aβ plaques per area in the cortex was also significantly reduced in vatalanib-administered 5xFAD mice. However, the average size of Aβ plaques was not altered by vatalanib administration in 5xFAD mice (Fig. 1k).

In summary, we evaluated the effects of vatalanib on AD pathology in 5xFAD mice. Previous studies have demonstrated that both AT8- and AT100-labeled phospho-tau epitopes are elevated in the AD brain [13, 14]. In addition, 5xFAD mice have been reported to exhibit hyperphosphorylation of tau and decreased p-GSK-3β (Ser9) [15]. In the present study, administration of vatalanib attenuated tau phosphorylation at AT8 and AT100 in 5xFAD mice, and these changes were accompanied by increased GSK-3β^Ser9^ phosphorylation (Fig. 1c–i). These results indicate that vatalanib differentially regulates tau phosphorylation by modulating the GSK-3β phosphorylation epitope in 5xFAD mice. While vatalanib administration had no effect on Aβ fluorescence intensity or Aβ plaque size, the number of Aβ plaques excluding intracellular Aβ in the cortex was significantly decreased in 5xFAD mice (Fig. 1k). Considering that 3- to 4-month-old 5xFAD mice predominantly accumulate Aβ in the deep cortical layer and subiculum and not the hippocampus, it is reasonable that vatalanib has an inhibitory effect on Aβ accumulation in the cortex [16]. It is possible that long-term administration of vatalanib might inhibit Aβ accumulation according to age. Interestingly, a recent study found that Aβ oligomers activate microglial cells in a tyrosine kinase-dependent manner [17]. In addition, since VEGF and VEGFR are involved in BBB permeability and microglial chemotaxis, future studies will address whether vatalanib affects neuroinflammatory responses and BBB integrity in 5xFAD mice [7–9]. Moreover, we will evaluate the effects of vatalanib on tauopathy, basal behavior, and cognitive impairment in a mouse model of AD (e.g., PS19 Tau Tg or 5xFAD mice) and identify the mechanism by which vatalanib alters tau phosphorylation as well as synaptic function. In conclusion, intraperitoneal injection of vatalanib in 3-month-old 5xFAD mice reduced Aβ plaque levels and tau phosphorylation, and thus vatalanib may be a drug candidate for Ab-and/or tau-associated diseases, including AD.

## Supplementary information

**Additional file 1.:** Materials and methods.

## Data Availability

The datasets generated during and/or analyzed during the current study are available from the corresponding author on reasonable request.

## Abbreviations

Aβ: Amyloid β
AD: Alzheimer’s disease
APP: Amyloid precursor protein
CDK-5: Cyclin-dependent kinase-5
DG: Dentate gyrus
GSK-3β: Glycogen synthase kinase-3β
NFT: Neurofibrillary tangle
PDGFRα: Platelet-derived growth factor receptor α
TKI: Tyrosine kinase inhibitor
VEGF: Vascular endothelial growth factor
VEGFR: Vascular endothelial growth factor receptor
