# Supplementary material for: The VEGF inhibitor vatalanib regulates AD pathology in 5xFAD mice
Source: Mol Brain. 2020 Sep 25;13:131. doi: 10.1186/s13041-020-00673-7 (PMC7519542; doi:10.1186/s13041-020-00673-7)
Supplement: Supplementary file 1 — Additional file 1.: Materials and methods. [file 13041_2020_673_MOESM1_ESM.docx]

**Materials and methods**

**Animals**

Female B6SJLF1/J mice (JAX stock #100012, The Jackson Laboratory, Bar Harbor, ME, USA) and male 5xFAD mice (B6SJL-Tg(APP^SwFlLon^,PSEN1*M146L*L286V)6799Vas/Mmjax) (MMRRC stock #34840, The Jackson Laboratory, Bar Harbor, ME, USA) were crossed, and the first generation was used in the experiment. All animals were housed at 22°C with a 12-hour day: night cycle in the SPF facility of the Korea Brain Research Institute (KBRI). All animal experiments were performed in accordance with approved animal protocols and guidelines established by the institutional animal care and use committee of KBRI (IACUC-2016-0013, IACUC-19-00049, IACUC-19-00042).

**Drug administration**

Vatalanib (Cat #A3969, ApexBiO, Houston, TX, USA) was prepared in vehicle (18% PEG + 5% Tween 80 + 2% DMSO). Three-month-old male 5xFAD mice were intraperitoneally injected with 10 mg/kg vatalanib or vehicle daily for 14 days; no significant weight loss or morbidity occurred during this period. Drug administration and histopathological analysis were performed by different researchers. For each experiment, two independent researchers confirmed the quantification results, but animals were not injected in a blinded manner.

**Brain section preparation**

5xFAD mice were fixed by sequential cardiac perfusion with phosphate-buffered saline (PBS) and 4% paraformaldehyde (PFA) solution. Brains extracted from the 5xFAD mice were post-fixed with 4% PFA solution for 20 hours at 4°C and subsequently cryoprotected with 30% sucrose solution for 3 days at 4°C. The cryoprotected brains were mounted with optimal cutting temperature (OCT) compound (Tissue-Tek, Sakura Finetek, Torrance, CA, USA) and sectioned into a 35-μm-thick coronal plane with a Leica CM1860 cryostat (Leica Biosystems, Buffalo Grove, IL, USA).

**Immunohistochemistry**

Free-floating brain sections (2 slices from one animal) collected from -1.70 to -2.30 mm relative to the bregma in stereotaxic coordinates were incubated overnight at 4°C in PBS containing 0.3% Triton X-100 and 0.5 mg/mL BSA with one of the following primary antibodies: anti-AT8 (1:1000; Cat #mn1020, Invitrogen, Carlsbad, CA, USA), anti-AT100 (1:1000; Cat #mn1060, Invitrogen), anti-Tau-5 (1:1000; Cat #AHB0042, Invitrogen), anti-p-GSK-3β^Ser9^ (1:1000; Cat #9323S, Cell Signaling Technology, Danvers, MA, USA), anti-p-CDK5^Tyr15^ (1:1000; Cat #LS‑C354604, LSbio, Seattle, WA, USA), or anti-6E10 (1:1000; Cat #SIG-39320, BioLegend, San Diego, CA, USA). The sections labeled with the primary antibody were washed 3 times with PBS and then incubated under free-floating conditions with Alexa Fluor 488- or 555-conjugated anti-rabbit or anti-mouse secondary antibody (1:1000; Invitrogen) for 1 hour at room temperature. Finally, the immunofluorescently stained sections were washed 3 times with PBS and mounted on pre-washed glass slides with VECTASHIELD Antifade Mounting Medium with DAPI (Cat #H-1200, Vector Laboratories Burlingame, CA, USA). Two sections per animal were processed for immunofluorescence staining, and the left and right hemispheres were photographed with a Leica DMI8 inverted fluorescence microscope (Leica Microsystems, Wetzlar, Germany).

**Histological quantification**

Levels of AT100, AT8, Tau5, p-GSK3β and p-CDK5 fluorescence in the cortex and hippocampal CA1 and DG regions were quantified by first drawing a region of interest (ROI) using ImageJ software as previously described [1, 2] on an image with DAPI fluorescence. Red (Tau-5) or green (AT100, AT8, p-GSK3β or p-CDK5) fluorescence intensity was analyzed by overlaying the ROIs on the images, and AT100, AT8, Tau-5, p-GSK3β and p-CDK5 expression were quantified by dividing the intensity level by the ROI area. The percentage of immunopositive area in the total area was calculated after classifying the immunopositive area through thresholding. The particle analysis function of ImageJ software (version 1.53a, U. S. National Institutes of Health, Bethesda, ME, USA) was used to count and calculate the number of Aβ plaques per area and their average size after excluding intracellular Aβ.

**Statistical analyses**

All histological quantification results of the vatalanib-treated group were normalized by the vehicle-treated group. All data were analyzed using a two-tailed unpaired *t*-test with GraphPad Prism 7 software (GraphPad Software, San Diego, CA. USA). Data are presented as the mean ± S.E.M. (**p* < 0.05, ***p* < 0.01, ****p* < 0.001).

**References**

1. Lee JY, Nam JH, Nam Y, Nam HY, Yoon G, Ko E, Kim SB, Bautista MR, Capule CC, Koyanagi T, et al: **The small molecule CA140 inhibits the neuroinflammatory response in wild-type mice and a mouse model of AD.** *J Neuroinflammation* 2018, **15:**286.

2. Ryu KY, Lee HJ, Woo H, Kang RJ, Han KM, Park H, Lee SM, Lee JY, Jeong YJ, Nam HW, et al: **Dasatinib regulates LPS-induced microglial and astrocytic neuroinflammatory responses by inhibiting AKT/STAT3 signaling.** *J Neuroinflammation* 2019, **16:**190.
